# Potential Role of Soluble Metal Impurities in the Acute Lung Inflammogenicity of Multi-Walled Carbon Nanotubes

**DOI:** 10.3390/nano10020379

**Published:** 2020-02-21

**Authors:** Dong-Keun Lee, Soyeon Jeon, Jiyoung Jeong, Il Je Yu, Kyung Seuk Song, Aeyeon Kang, Wan Soo Yun, Jong Sung Kim, Wan-Seob Cho

**Affiliations:** 1 Lab of Toxicology, Department of Medicinal Biotechnology, College of Health Sciences, Dong-A University, Busan 49315, Korea; dnjsxo356@naver.com (D.-K.L.); wjsthdus0418@naver.com (S.J.); dudwlwjd@naver.com (J.J.); 2 HCTm Co., LTD., 74, Seoicheon-ro 578 beon-gil Majang-myeon, Icheon-si, Gyeonggi-do 17383, Korea; u1670916@chol.com; 3 Korea Conformity Laboratories, 8, Gaetbeol-ro 145 beon-gil, Yeonsu-gu, Incheon 21999, Korea; songks@kcl.re.kr; 4 Department of Chemistry, Sungkyunkwan University, 2066, Seobu-ro, Jangan-gu, Suwon-si, Gyeonggi-do 16419, Korea; aeyeon1028@naver.com (A.K.); wsyun87@skku.edu (W.S.Y.); 5 Department of Community Health and Epidemiology, Dalhousie University, Halifax, NS B3H4R2, Canada; jskim@Dal.Ca

**Keywords:** inflammation, lung, metal impurities, multi-walled carbon nanotubes, soluble fraction, transitional metals

## Abstract

Multi-walled carbon nanotubes (MWCNTs) have variable metal impurities, but little is known about the impact of soluble metal impurities on the toxicity of MWCNTs. Here, we evaluated the role of soluble metal impurities to the acute inflammogenic potential of MWCNTs, using five types of high purity MWCNTs (>95%). MWCNTs and their soluble fractions collected at 24 h after incubation in phosphate-buffered saline showed diverse metal impurities with variable concentrations. The fiber-free soluble fractions produced variable levels of reactive oxygen species (ROS), and the iron level was the key determinant for ROS production. The acute inflammation at 24 h after intratracheal instillation of MWCNTs to rats at 0.19, 0.63, and 1.91 mg MWCNT/kg body weight (bw) or fiber-free supernatants from MWCNT suspensions at 1.91 and 7.64 mg MWCNT/kg bw showed that the number of granulocytes, a marker for acute inflammation, was significantly increased with a good dose-dependency. The correlation study showed that neither the levels of iron nor the ROS generation potential of the soluble fractions showed any correlations with the inflammogenic potential. However, the total concentration of transition metals in the soluble fractions showed a good correlation with the acute lung inflammogenic potential. These results implied that metal impurities, especially transitional metals, can contribute to the acute inflammogenic potential of MWCNTs, although the major parameter for the toxicity of MWCNTs is size and shape.

## 1. Introduction

The toxicity of multi-walled carbon nanotubes (MWCNTs) is closely related to their physicochemical properties, such as size, shape, solubility, and rigidity [1,2]. As these properties depend on the methods of manufacture, the toxicity study of specific MWCNTs cannot represent all MWCNTs. Therefore, the read-across study to evaluate the physicochemical parameters related to the toxicity endpoints of MWCNTs represents a promising approach to help establish the regulatory requirements [3,4,5]. Recent studies have suggested the biopersistent, long, and rigid fibers are more pathogenic than dissolving, short, or tangled fibers, because these physical parameters are related to frustrated phagocytosis, which is the major pathogenicity paradigm for asbestos fibers [2,6,7,8].

Unlike the physical properties, the chemical properties of MWCNTs are not well defined in relation to the toxicity endpoints. The chemical properties of MWCNTs include elemental composition, impurities, surface charge, and potential for quenching/generating radicals [9]. In the absence of surface modifications, the surface of pure MWCNTs without defects is similar, because MWCNTs are simply composed of hexagonal graphite sheets [10]. Pure hexagonal graphite-based materials without defects are generally less reactive and have a high potential for quenching free radicals than metal/metal oxide nanoparticles; therefore, pure carbon materials can be classified as a low toxicity group if their physical properties are not considered [11,12,13]. However, 100% pure MWCNTs are hardly prepared as a final product because of the addition of metal catalysts during synthesis processes [14].

The purity of MWCNTs available in the market varies considerably between 50% and 99.9%, depending on the purification processes post-synthesis [15,16]. More than 20 metals or metalloids have been reported as contaminants in MWCNTs; the majority are transition metals, such as iron, nickel, molybdenum, yttrium, cobalt, and chromium [17]. Metal impurities have been considered to be an important factor in the risk assessment of MWCNTs because they may contribute to the toxicity of MWCNTs in respect to toxicological and immunological effects [9,18,19]. However, few studies have investigated the effect of metal impurities on the toxicity endpoints of MWCNTs, because the physical property of MWCNTs and its similarity to asbestos fibers, such as size and shape, is the major parameter of the fiber pathogenicity paradigm. In this study, we evaluated the impact of soluble metal impurities on the acute lung inflammogenic potential of MWCNTs and their mechanism of action.

## 2. Materials and Methods

### 2.1. Panel of MWCNT and Evaluation of Physical Properties

Five different types of MWCNTs were prepared and designated as CNT1 to CNT5, respectively: CNT1 (Kumho Petrochemical Co., Daejeon, Korea), CNT2 (Neone Co., Seoul, Korea), CNT3 (JEIO Co., Incheon, Korea), CNT4 (Hanwha Nanotech Co., Seoul, Korea), and CNT5 (Mitsui & Co., Tokyo, Japan). CNT5, which is also called Mitsui-7, is a reference material and is classified as a Group 2B carcinogen by the International Agencies for Research on Cancer (IARC) (IARC, 2017). The size and shape of MWCNTs were evaluated, using scanning electron microscope (SEM) (JSM-6700F; JEOL, Tokyo, Japan) and transmission electron microscope (TEM) (JEM-1200EX II, JEOL, Tokyo, Japan). Briefly, MWCNTs were dispersed at 1 mg/mL with heat-inactivated fetal bovine serum (FBS) in distilled water (DW) and sonicated for 1 h in a bath sonicator (Saehan-Sonic, Seoul, Korea). Then, a final working solution of 100 μg/mL was produced by the addition of DW and subjected to 30 min sonication in the bath sonicator. The working solution was washed twice by centrifugation at 15,000 g for 15 min, to remove unbound serum components, and then 100 μL (10 μg MWCNTs) was placed on the SEM filter (Merck Millipore Ltd., Cork, Ireland) and dried overnight at room temperature. For TEM, 10 μL of the working solution was dried on a copper grid (Electron Microscopy Sciences, Hatfield, PA, USA) at room temperature. The mean diameter and lengths of MWCNTs were measured by counting at least 300 fibers, using a built-in analysis program (JEOL). The rigidity of MWCNTs was measured by using TEM in accordance with the International Standard Organization standard method (ISO/TS 11888:2017) [20]. The surface area of MWCNTs was measured by the Brunauer–Emmett–Teller method, using a BELSORP-mini II (BEL Japan Inc., Toyonaka, Japan). The defects in MWCNTs were evaluated by Raman spectroscopy, using a WITec alpha300 system (WITec GmbH, Ulm, Germany) with incident laser light at 532 nm. The levels of endotoxins in MWCNTs were evaluated by a colorimetric Limulus amoebocyte lysate (LAL) assay kit (Cambrex; Walkersville, MD, USA), at the highest concentration used in the animal study (600 μg/mL in DW).

### 2.2. Evaluation of Chemical Properties

#### 2.2.1. Purity and Metal Impurities of MWCNT Powders

The purity of MWCNTs was measured by using thermogravimetric analysis (TGA) and inductively coupled plasma-mass spectrometry (ICP-MS). TGA and differential thermal analyses (DTA) were performed by using a Seiko Exstar 7300 TG/DTA7300 unit (Seiko Instrument Inc., Tokyo, Japan), under a nitrogen atmosphere, at a heating rate of 10 °C/min, up to 1020 °C. To evaluate the metal impurities of MWCNT powders, 3 mL of 60% HNO_3_ was mixed with 0.1–0.2 g MWCNTs and heated for 6 h at 200 °C. Elemental concentrations were measured by using an Agilent 7500 (Agilent; Santa Clara, CA, USA) for 6 h. The purity and impurities of CNT5 were also compared with previous publications [21,22].

#### 2.2.2. Soluble Metal Impurities of MWCNTs

To evaluate the soluble metal impurities of MWCNTs, fibers were dispersed and incubated for 24 h in phosphate-buffered saline (PBS; Sigma-Aldrich, St. Louis, MO, USA) or artificial lysosomal fluid (ALF), which was used to mimic interstitial fluid or lysosomal fluid, respectively. To prepare ALF, 55 mM of NaCl, 150 mM of NaOH, 108 mM of citric acid, 0.87 mM of CaCl_2_, 0.67 mM of Na_2_HPO_4_·7H_2_O, 0.27 mM of Na_2_SO_4_, 0.52 mM of MgCl_2_·6H_2_O, 0.64 mM of glycerin, 0.26 mM of sodium citrate dehydrate, 0.39 mM of sodium tartrate dihydrate, 0.76 mM of sodium lactate, 0.78 mM of sodium pyruvate, and 1 mL of formaldehyde were mixed, and the pH was adjusted to 5.5 [23] (Stopford et al., 2003). MWCNTs were dispersed in PBS or ALF at 100 μg/mL, sonicated in a bath sonicator for 1 h, and incubated for 24 h at room temperature. Ultracentrifugation at 50,000 rpm for 3 h was performed, using an Optima AUC (Beckman Coulter; Indianapolis, IN, USA) to collect the MWCNT-free supernatants, and the concentration of metals was analyzed by using an Agilent 7700x ICP-MS (Agilent, Santa Clara, CA, USA). SEM was used to confirm the absence of MWCNT contamination in the supernatant. To prepare for SEM observations, 100 μL of undiluted supernatant was placed on the SEM filter (Merck Millipore Ltd., Burlington, MA, USA) and dried overnight at room temperature.

### 2.3. Evaluation of Reactive Oxygen Species (ROS) Generation Potentials of MWCNTs or MWCNT-Free Supernatants

To evaluate the potential for ROS generation, the cell-free 2′7′-dichlorofluorescein diacetate (DCFH-DA) assay was applied according to previously described methods [24]. Briefly, 5 mM of DCFH-DA (Calbiochem; La Jolla, CA, USA) in ethanol was mixed with 0.01 N sodium hydroxide at a ratio of 1:40 and incubation for 30 min at room temperature. Then, the mixed solution was neutralized by the addition of 25 mM of PBS (pH 7.2) at a ratio of 1:200 and kept on ice, followed by the addition of 2.2 U/mL of horseradish peroxidase (Sigma-Aldrich, St. Louis, MO, USA). The suspensions or MWCNT-free supernatants from various concentrations of MWCNTs (3–100 μg/mL) in 10 mM PBS (pH 7.4) was mixed 1:1 with DCFH-DA solution and incubated at 37 °C for 15 min. To exclude the optical interference by MWCNTs, the mixtures were centrifuged at 15000 × g for 15 min, and fluorescence intensity was measured at 485/590 nm, using a Synergy HT Multi-Mode Microplate Reader (Bio-Tek Instruments; Winooski, VT, USA). The hydrogen peroxide (H_2_O_2_, Sigma-Aldrich, St. Louis, MO, USA) at 0–10 μM was used for the standard curve, and the levels of ROS generation were expressed as H_2_O_2_ equivalent.

### 2.4. Preparation of MWCNTs or MWCNT-Free Soluble Fractions for In Vivo Experiments

MWCNTs were dispersed as described previously [7]. Briefly, a stock solution of MWCNTs was dispersed at 1 mg/mL in 30% heat-inactivated rat serum and sonicated for 1 h in a bath sonicator. Then, PBS was added to obtain the working concentration (i.e., 60, 200, and 600 μg/mL). The concentration of rat serum in the working solution was less than 3%. To evaluate the effect of soluble fraction, the MWCNT-free soluble fractions from MWCNTs in PBS (pH 7.4) were instilled into the lungs of rats. Briefly, the working solutions of MWCNTs at 600 and 1200 μg/mL were incubated for 24 h at room temperature, and MWCNT-free supernatants were collected by ultracentrifugation at 50,000 rpm for 3 h.

### 2.5. Intratracheal Instillation of MWCNTs or MWCNT-Free Soluble Fractions

Six-week-old specific-pathogen-free female Wistar rats (Samtako, Gyeonggi-Do, Korea) were purchased and acclimatized for one week before the experiment. The rats were maintained and handled according to policies approved by the Institutional Animal Care and Use Committee of Dong-A University. The rats were housed in a micro ventilation cage system (MVCS; Three Shine Inc.; Daejeon, Korea), under controlled conditions, at 22 ± 1 °C, 50 ± 10% humidity, and 12 h light/dark cycle. Intratracheal instillation was performed according to previously described methods [25]. Briefly, rats were anesthetized with isoflurane (Piramal Critical Care; Bethlehem, PA, USA), using a rodent anesthesia system (VetEquip; Pleasanton, CA, USA). Then, a 16-gauge polycarbonate catheter was intubated into the trachea, and the suspensions of MWCNTs or soluble fractions were instilled at a volume of 500 μL/rat. PBS with 3% rat serum was used for vehicle control (VEH). The treatment doses for MWCNTs were 1.19, 0.63, and 1.91 mg of MWCNT/kg body weight (bw), which corresponded to the 30, 100, and 300 µg/rat (the average bw of rats was 156.99 g). The treatment doses for soluble fractions of MWCNTs can be converted to the equivalent dose for 1.91 and 7.64 mg MWCNT/kg bw.

### 2.6. Preparation of Bronchoalveolar Lavage Fluid (BALF)

At 24 h after instillation, the acute lung inflammogenic potential was evaluated by the BALF analysis. Rats were sacrificed by removing blood from the inferior vena cava under deep isoflurane anesthesia. The lung was lavaged in situ, 4 times, using 8 mL of cold sterile Ca^2+^- and Mg^2+^-free PBS (Life Technologies; Gaithersburg, MD, USA), after cannulating by using a 14-gauge blunt stainless-steel needle. Then, BALF was centrifuged at 2000 *g* for 5 min, and the supernatant of the first lavage was kept separately for biochemical analysis. Cell pellets from 4 lavages were pooled and counted, using a NucleoCounter (Chemometec; Allerod, Denmark). Then, 4 × 10^4^ cells were attached onto the glass slides by cytospin centrifugation (Hanil; Incheon, Korea). The slides were then stained with Diff-Quick (Thermo Fisher Scientific; Waltham, MA, USA), and differential cell counting was performed by counting at least 300 nucleated cells under a light microscope based on the morphology of cells.

### 2.7. Measurement of Lactate Dehydrogenase (LDH) and Total Protein in BALF

As a marker for cytotoxicity, the levels of LDH were measured in BALF, using an LDH assay kit (Roche Diagnostics; Mannheim, Germany) according to the instruction manual. The levels of total protein, a marker for vascular permeability, were measured in BALF, using a bicinchoninic acid (BCA) assay kit (Thermo Fisher Scientific, Waltham, MA, USA).

### 2.8. Statistical Analysis

All graph and statistical analyses were performed using GraphPad Prism software (ver. 6.0; La Jolla, CA, USA). Data are presented as the mean ± standard error of the mean. Each group was compared by one-way analysis of variance (ANOVA) with post hoc Tukey’s pairwise comparisons. The Spearman correlation test was applied to evaluate parameters producing ROS generation or lung inflammation. A *p*-value of less than 0.05 was considered statistically significant.

## 3. Results

### 3.1. Physical Characteristics of MWCNTs

**Figure 1:**
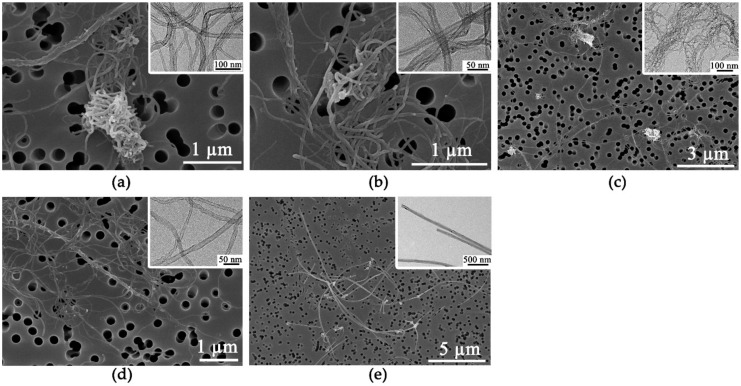
SEM and TEM images of MWCNTs. The large square images are SEM images, and the small insert square images are TEM images. CNT1 (**a**), CNT2 (**b**), CNT3 (**c**), and CNT4 (**d**) showed tangled form, while CNT5 (**e**) showed straight form.

The physical characteristics of the prepared MWCNTs are presented in Table 1. All MWCNTs were long fibers longer than 1 µm. The SEM and the TEM images showed that four MWCNTs (CNT1 –CNT4) were in a tangled form, whereas those of type CNT5 were straight (Figure 1). In addition, the diameter of the tangled MWCNTs was thinner than that of the straight form. The surface area of MWCNTs was closely correlated with the diameter, implying that thinner MWCNTs had a larger surface area (Figure S1, Supplementary Materials). The IG/ID ratio from Raman spectroscopy showed a variety of defects in the MWCNTs, but these were not specifically correlated with other physicochemical properties. The levels of endotoxin contamination in the MWCNTs suspensions were below the limit of detection (<0.1 EU/mL).

### 3.2. Chemical Characteristics of MWCNTs

The purity data obtained by TGA showed that all MWCNTs were more than 90% pure (Table 2). Meanwhile, the ICP-MS data showed that all MWCNTs were more than 95% pure, with aluminum, iron, and cobalt identified as common metal impurities (Table 2). Soluble metal impurities were measured under two different conditions that mimicked the in vivo conditions pre- and post-phagocytosis. The metal impurities of the soluble fractions of MWCNTs dissolved in PBS contained more than 20 elements at ppb levels (Table 3). Meanwhile, the concentrations of selected elements (e.g., cobalt, iron, and nickel) in the soluble fractions in ALF were much higher than those dispersed in PBS (Table S1, Supplementary Materials).

### 3.3. ROS Generation Potential of MWCNTs or Soluble Fractions

**Figure 2:**
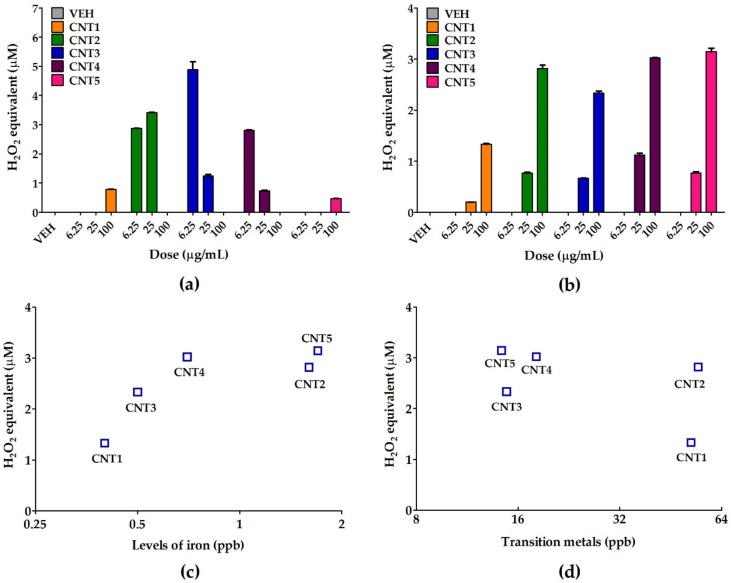
The ROS generation potential of MWCNTs or soluble fractions. A cell-free DCFH-DA assay was used to measure the ROS generation potential of (**a**) MWCNT or (**b**) the MWCNT-free soluble fractions. Plots of the concentration of (**c**) iron or (**d**) transition metals against the ROS generation potentials of the MWCNT-free soluble fractions at 100 µg/mL of MWCNT suspensions. Iron was the highly correlated element for ROS generation (Spearman correlation coefficient *r* = 0.90). VEH, vehicle control; *n* = 4 for ROS measurement.

The ROS generation potential of MWCNTs or soluble fractions was evaluated by the cell-free DCFH-DA assay. The absence of fibers in the soluble fractions was confirmed by SEM (Figure S2, Supplementary Materials). MWCNTs resulted in increased ROS generation, but there was no consistent dose-dependence (Figure 2a). In contrast, the ROS generation potentials of the soluble fraction of MWCNTs showed a strong ROS generation potential for all MWCNTs, with an excellent dose-dependency (Figure 2b). A plot of the metal impurities against the levels of ROS showed that, among the various metal impurities, the concentration of iron was closely correlated with the potential for ROS generation (Spearman correlation coefficient *r* = 0.90), whereas the correlation of transitional metals yielded no specific pattern (Figure 2c,d).

### 3.4. Acute Lung Inflammation by MWCNTs

**Figure 3:**
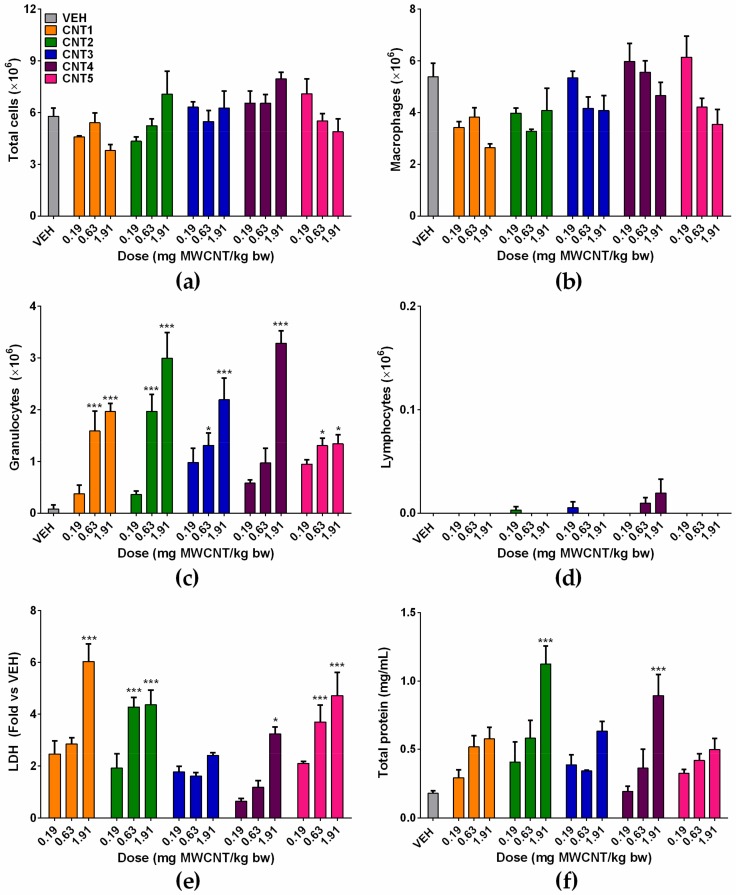
The acute lung inflammation at 24 h after intratracheal instillation of MWCNTs to rats. MWCNTs were instilled at 30, 100, and 300 μg/rat, and cytological and biochemical analysis of BALF were performed. (**a**) The number of total cells; (**b**) the number of alveolar macrophages; (**c**) the number of granulocytes; (**d**) the number of lymphocytes; (**e**) the levels of LDH; and (**f**) the concentration of total protein in BALF. ^*^ *p* < 0.05, ^**^ *p* < 0.01, and ^***^ *p* < 0.001 compared with the vehicle control (VEH); *n* = 4 per group.

The acute inflammation of MWCNTs was evaluated by cytological and biochemical analyses of BALF at 24 h after intratracheal instillation of MWCNTs. The number of total cells showed no significant increases in any treatment groups, compared to the vehicle control group (Figure 3a). The number of macrophages and lymphocytes was comparable to the vehicle control group, while the number of granulocytes increased significantly by the treatment of MWCNTs compared to the vehicle control group in a dose-dependent manner (Figure 3b–d). The significant increases of granulocytes in BALF were observed at 0.63 and 1.91 mg MWCNT/kg bw for CNT1, CNT2, CNT3, and CNT5, and at 1.91 mg MWCNT/kg bw for CNT4 (Figure 3c). The concentrations of LDH and total protein showed a dose-dependent increase in all MWCNTs (Figure 3e,f). However, the statistically significant increase of the LDH levels was observed by the treatment of CNT1, 2, 4, and 5 (Figure 3e). Meanhile, the concentration of total protein showed a significant increase in CNT2 and 4 (Figure 3f).

### 3.5. Effect of Soluble Metal Impurities on the Acute Inflammogenic Potential of MWCNTs

**Figure 4:**
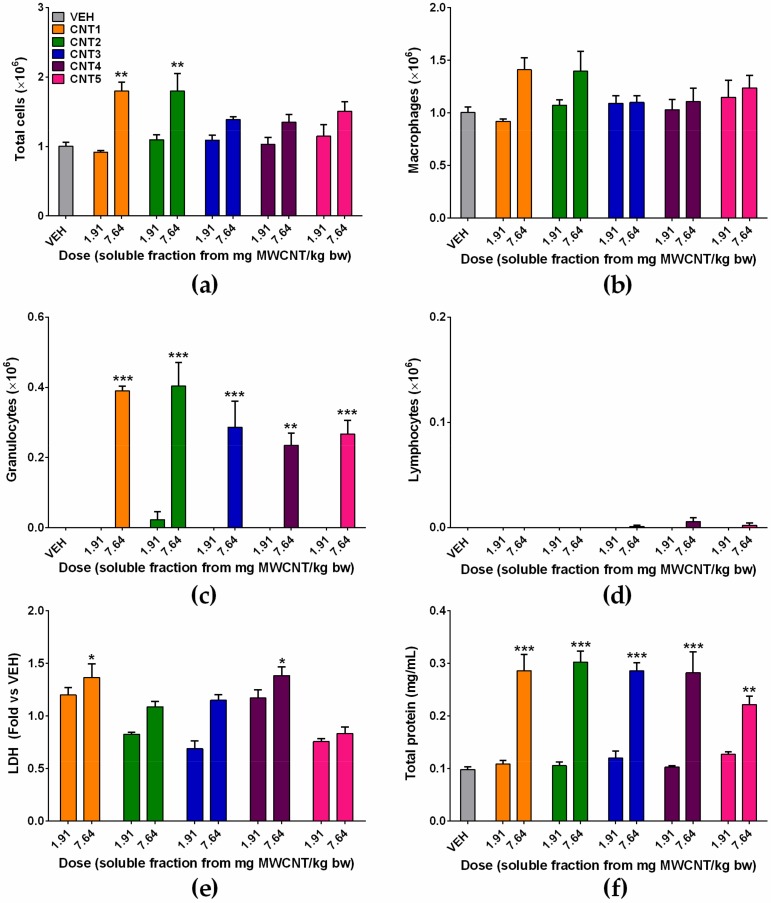
The acute pulmonary inflammation at 24 h after intratracheal instillation of the MWCNT-free soluble fractions to rats. The MWCNT-free soluble fractions were collected by ultracentrifugation of suspensions of MWCNTs at equivalent doses of 0.3 and 1.2 mg/rat, and cytological and biochemical analyses were performed in BALF. (**a**) The number of total cells; (**b**) the number of alveolar macrophages; (**c**) the number of granulocytes; (**d**) the number of lymphocytes; (**e**) the levels of LDH; and (**f**) the concentration of total protein. ^*^ *p* < 0.05, ^**^ *p* < 0.01, and ^***^ *p* < 0.001 compared with the vehicle control (VEH); *n* = 4 per group.

To evaluate the impact of soluble metal impurities on the magnitude of acute inflammation by MWCNTs, the soluble fractions collected by ultracentrifugation of the suspensions of MWCNTs in PBS were instilled into the lungs of rats. Because the treatment of MWCNT at 1.91 mg MWCNT/kg bw produced only neutrophilic inflammation without influencing the number of total cells and macrophages, the effect of soluble fractions of MWCNTs on the inflammation was tested at a fourfold higher dose of MWCNT (i.e., 7.64 mg MWCNT/kg bw). The number of total cells was significantly increased by the treatment of soluble fraction from 7.64 mg MWCNT/kg bw of CNT1 and 2, while other treatment groups showed no significant changes (Figure 4a). The number of macrophages and lymphocytes showed no significant changes (Figure 4b,d). While the number of granulocytes was significantly increased at the high dose of soluble fractions of all types of MWCNTs (Figure 4c). However, the low dose of soluble fraction collected from the suspension of MWCNT at 1.91 mg MWCNT/kg bw showed no inflammation, which implies that the dose required to produce acute neutrophilic inflammation by the soluble fraction should be much higher than that required for MWCNTs. The levels of LDH were significantly increased by the treatment of soluble fraction from 7.64 mg MWCNT/kg bw of CNT1 and 4 (Figure 4e). The concentration of total protein in BALF was significantly increased at the high dose of soluble fractions of all types of MWCNTs (Figure 4f).

### 3.6. The Parameter Triggering the Acute Lung Inflammation of Soluble Fractions

**Figure 5:**
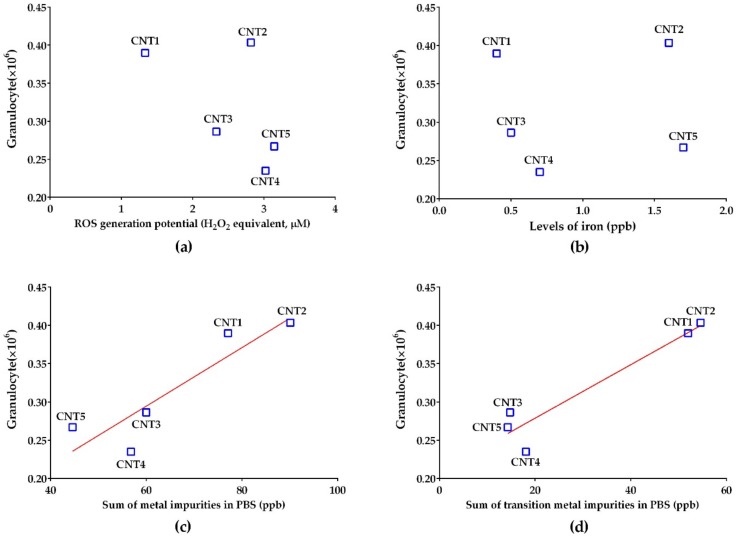
Correlation plots to evaluate the main parameters of the soluble fractions producing acute pulmonary inflammation. Plots of (**a**) ROS levels or (**b**) iron concentration against the number of granulocytes showed a poor correlation, but plots of (**c**) total metal impurities or (**d**) total transition metal impurities against the number of granulocytes showed good correlation (Spearman *r =* 0.9 and 0.7, respectively). The doses used for plotting of ROS generation potential and lung inflammation were 100 μg/mL and soluble fraction from 7.64 mg MWCNT/kg bw, respectively.

To evaluate the chemical parameter triggering the acute lung inflammation of soluble fractions, the number of granulocytes in BALF was plotted against the ROS generation potentials or ICP-MS data of the soluble fractions. Unlike the excellent correlation between the levels of ROS and iron, the acute lung inflammation data of the soluble fractions were not well correlated with any factors (Figure 5a,b). However, the number of granulocytes was well correlated with the total concentration of metals and metalloids in the soluble fractions (Spearman correlation coefficient *r* = 0.90; Figure 5c). Among all the metals and metalloids components, the concentrations of transition metals showed an excellent correlation coefficient (Spearman *r* = 0.70; Figure 5d). Meanwhile, the number of granulocytes showed poor correlations with the data of metal impurities in ALF, either with single elements or combinations (data not shown).

## 4. Discussion

MWCNTs can induce asbestos-like toxicities, owing to their physical properties, such as high biopersistence, length of >5 μm, an aspect ratio of >3:1, and high rigidity (D_b_ > 0.97 and SBPL > 1.08) [7,8]. In contrast to their physical properties, the role of chemical properties to the toxicity of MWCNTs is poorly understood. Metal impurities are a major factor affecting the chemical properties of MWCNTs and can influence various parameters, such as surface reactivity and ROS generation potential [9]. In this study, we hypothesized that metal impurities contribute to the inflammogenic potential of MWCNTs, even when the MWCNTs are relatively pure (>95%). To evaluate this hypothesis, the acute inflammogenic potentials of MWCNTs were compared with those of MWCNT-free soluble fractions, and the physicochemical parameters that accompanied the inflammation were investigated.

The detection of more than 20 metals or metalloids in the soluble fractions of high purity (>95%) MWCNTs dispersed in PBS suggests that metal impurities can contribute to the toxicity of MWCNTs because the purity of MWCNTs on the market varies from 50% to 99.9% [16,17]. The major elements contaminated in the MWCNTs were variable by the conditions used for MWCNT preparation. For example, aluminum, cobalt, and iron were the major contaminant metals in MWCNT powders, whereas aluminum, arsenic, boron, molybdenum, and zinc were the five most common contaminants of the 21 elements identified when MWCNTs were dissolved in PBS. Furthermore, aluminum, iron, molybdenum, and nickel were the major elements identified when MWCNTs were dissolved in ALF. The high concentrations of arsenic, boron, molybdenum, and zinc in the MWCNTs dispersed in PBS may be due to the high solubility of the MWCNTs in PBS or water [26,27,28]. The elements found in the soluble fractions of MWCNTs were generally consistent with previous studies [13,29,30,31].

The ROS data from this study suggest that the major source of ROS generation by MWCNTs was not the fibers, but the dissolved metal impurities. Furthermore, iron was found to be the element responsible for ROS generation by the Fenton reaction [32,33]. The generation of ROS can be a pathogenic factor for metal oxide nanoparticles in vitro and in vivo [24,34,35,36]. In addition, iron has been suggested to be a causative factor producing the oxidative stress of nanomaterials or asbestos fibers [30,37,38]. In this study, we found that the iron is the critical element producing ROS, but the acute inflammogenic potential of soluble fractions was not well correlated with either the concentration of iron or the ROS generation potentials, suggesting that none was the main factor responsible for lung inflammation in rats induced by MWCNTs.

In this study, soluble metal impurities of MWCNTs partly contributed to the toxicity of MWCNTs. However, the inflammogenic potential of soluble fractions did not match with that of MWCNTs, suggesting that the soluble metals are not the main contributors to the acute pulmonary inflammation of MWCNTs. Likewise, soluble metals in ALF did not show any correlations with the inflammogenic potentials of MWCNTs, although concentrations of some of the toxic metals (e.g., iron, cobalt, and nickel) in ALF were much higher than those in PBS. The absence of correlations of metal impurities in ALF with the inflammogenic potential of soluble fractions in PBS could be because of the different compositions and concentrations of metal impurities by the conditions of the medium. In addition, the non-correlation of inflammogenic potential of MWCNTs and metal impurities in ALF could be because long MWCNTs used in this study are undergoing incomplete phagocytosis, thus fewer fibers are placed in the lysosomal fluid than short fibers.

The excellent correlation between the sum of metal impurities in the soluble fractions with the acute inflammogenic potential suggested that the acute lung inflammogenic potential of the soluble fractions originates from the combination of toxic metals, rather than one or two metal impurities. Furthermore, the excellent correlation of the sum of transition metals with the acute inflammogenic potential and the poor correlation of the total metal impurities, excluding transition metals with the acute inflammogenic potential, suggests that transition metals are the elemental group responsible for the acute lung inflammation induced by soluble fractions of MWCNTs. The effect of transition metals may be due to their specific properties, such as their catalytic and redox activities [39,40], but further studies are needed to understand the effect of metal impurities on the toxicity of MWCNTs.

Although metal impurities can influence the toxicity of MWCNT and are suggested to be one of the key physicochemical properties related to the toxicity of MWCNT, there is limited evidence on this issue [9]. Table 4 presents the literature about the role of metal impurities to the toxicity of MWCNT. These previous studies were focused on the toxic effect of metal catalysts contaminated in the MWCNTs. However, the effect of soluble metals on the toxicity of MWCNT is not reported elsewhere. In this study, we identified the hazard of soluble metal impurities and suggested that the iron level is closely related to the ROS generation potential, but it is not consistent with the magnitude of inflammation of the soluble fraction of MWCNT. Meanwhile the sum of metal impurities rather than one or two metal impurities was responsible for the inflammation of the soluble fraction of MWCNT.

## 5. Conclusions

Although the ROS generation of MWCNTs was produced by the iron, neither ROS nor iron levels showed any correlation with the inflammogenic potential of soluble fractions of MWCNTs or MWCNT itself. In contrast, the soluble transition metals were closely correlated with the inflammogenic potential of the soluble fraction of MWCNTs, suggesting that the soluble metal impurities contaminated to the MWCNT can contribute to the toxicity of MWCNTs, although the physical properties, such as size, shape, aspect ratio, and rigidity, are the major pathogenic factors for MWCNTs. The diverse soluble metal impurities and their possible contribution to the toxicity of high-purity MWCNTs (>95%) found in this study suggests that the metal impurities should be carefully considered in the toxicity of MWCNTs, as the purity of MWCNTs on the market varies from 50% to 99.9%.

## Figures and Tables

**Table 1:** Physical properties of MWCNTs.

MWCNTs	Diameter (nm)	Length (µm)	Rigidity		BET (m^2^/g)	Raman (IG/ID)
			D_b_	SBPL		
CNT1	16.37 ± 0.2	10–50 ^a^	0.49 ± 0.1	0.54 ± 0.0	218.26	0.85
CNT2	15.64 ± 0.1	1–25 ^a^	0.42 ± 0.0	0.48 ± 0.0	194.03	1.05
CNT3	7.75 ± 0.1	7.55 ^a^	0.47 ± 0.0	0.49 ± 0.0	675.44	0.64
CNT4	16.7 ± 0.2	3.55 ^a^	0.66 ± 0.0	0.86 ± 0.0	224.90	0.92
CNT5	58.3 ± 1.0	10.02 ± 0.3	0.99 ± 0.0	1.19 ± 0.0	28.2	1.01

^a^ The length data of CNT1–CNT4 were obtained from the manufacturers. D_b_, bending ratio; SBPL, static bending persistence length; BET, Brunauer–Emmett–Teller method.

**Table 2:** Purity and impurity data of MWCNT powders.

	CNT1	CNT2	CNT3	CNT4	CNT5
Purity (%) by TGA	>90	>95	>99	94.9	>99 ^a^
Purity (%) by ICP-MS	>95	>99	>99	>95	>99 ^b^
Major metal impurities (% by weight)	Fe: 0.84	Al: 0.12	Al: 0.15	Al < 4	Mg < 0.0002 ^b^
	Al: 0.74	Fe: 0.08	Mg: 0.14	Fe < 2	Al < 0.009 ^b^
	Co: 0.28	Cu: 0.01	Co: 0.05	Co < 2	Fe < 0.04 ^b^
	Ni: 0.0064		Cu: 0.01		Ni < 0.0001 ^b^

^a^ The TGA data of CNT5 was referred by Birch et al [21]. ^b^ The ICP-MS data of CNT5 was referred by Rahman et al. [22].

**Table 3:** Metal impurities (ppb) of soluble fractions of MWCNTs dispersed in PBS ^1^.

Metals	Property	CNT1	CNT2	CNT3	CNT4	CNT5
Al	Other metals	2.3	9.1	14.7	14.7	4.4
As	Metalloids	5.8	7.9	7.7	7.5	3.3
B	Metalloids	14.5	15.6	18	13.9	19.3
Ba	Alkaline metals	0.1	0.4	0.2	0.2	0.3
Co	Transition metals	5.5	6.1	2.8	5.3	0.0
Cr	Transition metals	0	0.1	0	0	0.1
Cu	Transition metals	0.1	0.4	0.2	0.1	0.2
Fe	Transition metals	0.4	1.6	0.5	0.7	1.7
Ga	other metals	0.1	0.1	0.1	0.1	0.1
Li	Alkali metals	0	0.2	0.2	0.1	0.3
Mn	Transition metals	0.1	0.2	0	0	0
Mo	Transition metals	35.9	35.9	0.2	0.2	0.1
Ni	Transition metals	0.2	0.2	0.4	0.1	0.2
Rb	Alkali metals	0.4	0.4	0.5	0.4	0.4
Sb	Metalloids	0.6	0.5	1.7	0.5	0.5
Se	Nonmetals	0.1	0.1	0.3	0.1	0.3
Sn	other metals	0.1	0.1	0	0.1	0
Sr	Alkaline metals	1.1	1.1	1.8	1.1	1.4
Ti	Transition metals	0.7	0.6	0.9	0.7	0.8
W	Transition metals	0.6	0.3	0.2	0.2	0.1
Zn	Transition metals	8.5	9.2	9.6	10.8	11.1

^1^ MWCNTs in PBS at 100 μg/mL were incubated for 24 h at room temperature, and MWCNT-free soluble fractions collected by ultracentrifugation at 50,000 rpm for 3 h. Note that Na, P, K, Si, Mg, and Ca were excluded, although the high concentrations were detected. The levels of Be, Cd, Ge, In, Nb, Pb, Re, Sc, Ta, Tl, V, Y, and Zr were not detected.

**Table 4:** The literature about the role of metal impurities to the toxicity of MWCNT.

Nanomaterials	Experimental Model	Toxicity	Reference
O-MWCNT (4.5% Ni, 0.8% Fe) P-MWCNT (1.8% Ni, 0.1% Fe) F-MWCNT (negligible Ni and Fe)	Instillation and inhalation to male SD rats	Inflammation was produced in the order of O-, P-, and F-MWCNT	[41]
Nine different types of MWCNT	Instillation	The magnitude of toxicity was strongly associated with the Ni contamination on the particle	[16]
O-MWCNT (4.5% Ni, 0.8% Fe) P-MWCNT (1.8% Ni, 0.1% Fe) F-MWCNT (negligible Ni and Fe)	In vitro: BEAS-2B, RLE-6TN, and THP-1	The levels of IL-1β in THP-1 cells were in the order of O-, P-, and F-MWCNT	[42]
Purified MWCNT (Co 0.07%, Fe 0.16%, Mg 0.05%) Unpurified MWCNT (Co 1.3%, Fe 2.4%, Mg 2.5%)	In vitro: Venous blood of healthy human volunteers, A549, and HaCaT	Unpurified MWCNT showed higher toxicity than purified MWCNT by increasing the oxidation reactions	[43]
Raw MWCNT (Fe 0.08%, Ni 2.2%) Purified MWCNT (Ni 0.96%)	In vitro: alveolar macrophages from C57BL/6 mouse, THP-1	Purified MWCNT showed less toxicity and inflammasome activation than raw MWCNT	[44]

## Supplementary Materials

The following are available online at https://www.mdpi.com/2079-4991/10/2/379/s1. Figure S1: The correlation plot between the diameter of MWCNTs and BET; **Figure S2**. The SEM image showing the absence of MWCNTs in the soluble fractions; Table S1: The levels of selected elements (ppb) of soluble fractions of MWCNTs dispersed in ALF (pH 5.5).
