# Supplementary material for: Potential Role of Soluble Metal Impurities in the Acute Lung Inflammogenicity of Multi-Walled Carbon Nanotubes
Source: Nanomaterials (Basel). 2020 Feb 21;10(2):379. doi: 10.3390/nano10020379 (PMC7075329; doi:10.3390/nano10020379)
Supplement: Supplementary file 1 [file nanomaterials-10-00379-s001.pdf]

Supplementary file

Potential Role of Metal Impurities in the Acute Lung Inflammogenicity of Multi-Walled Carbon Nanotubes

Dong-Keun Lee [1], Soyeon Jeon [1], Jiyoung Jeong [1], Il Je Yu [2], Kyung Seuk Song [3], Aeyeon Kang [4], Wan Soo Yun [4], Jong Sung Kim [5] and Wan-Seob Cho [1,*]

[1] Lab of Toxicology, Department of Medicinal Biotechnology, College of Health Sciences, Dong-A University, Busan 49315, Korea; dnjsxo356@naver.com (D.-K.L.); wjsthdus0418@naver.com (S.J.); dudwlwjd@naver.com (J.J.)

[2] HCTm Co., LTD., 74, Seoicheon-ro 578 beon-gil Majang-myeon, Icheon-si, Gyeonggi-do 17383, Korea; u1670916@chol.com (I.J.Y.).

[3] Korea Conformity Laboratories, 8, Gaetbeol-ro 145 beon-gil, Yeonsu-gu, Incheon 21999, Korea; songks@kcl.re.kr (K.S.S.).

[4] Department of Chemistry, Sungkyunkwan University, 2066, Seobu-ro, Jangan-gu, Suwon-si, Gyeonggi-do 16419, Korea; aeyeon1028@naver.com (A.K.); wsyun87@skku.edu (W.S.Y.).

[5] Department of Community Health and Epidemiology, Dalhousie University, Halifax, Nova Scotia B3H4R2, Canada; jskim@Dal.Ca (J.S.K.).

* Correspondence: wcho@dau.ac.kr; Tel.:+82-51-200-7563

Received: 30 January 2020; Accepted: 18 February 2020; Published: date

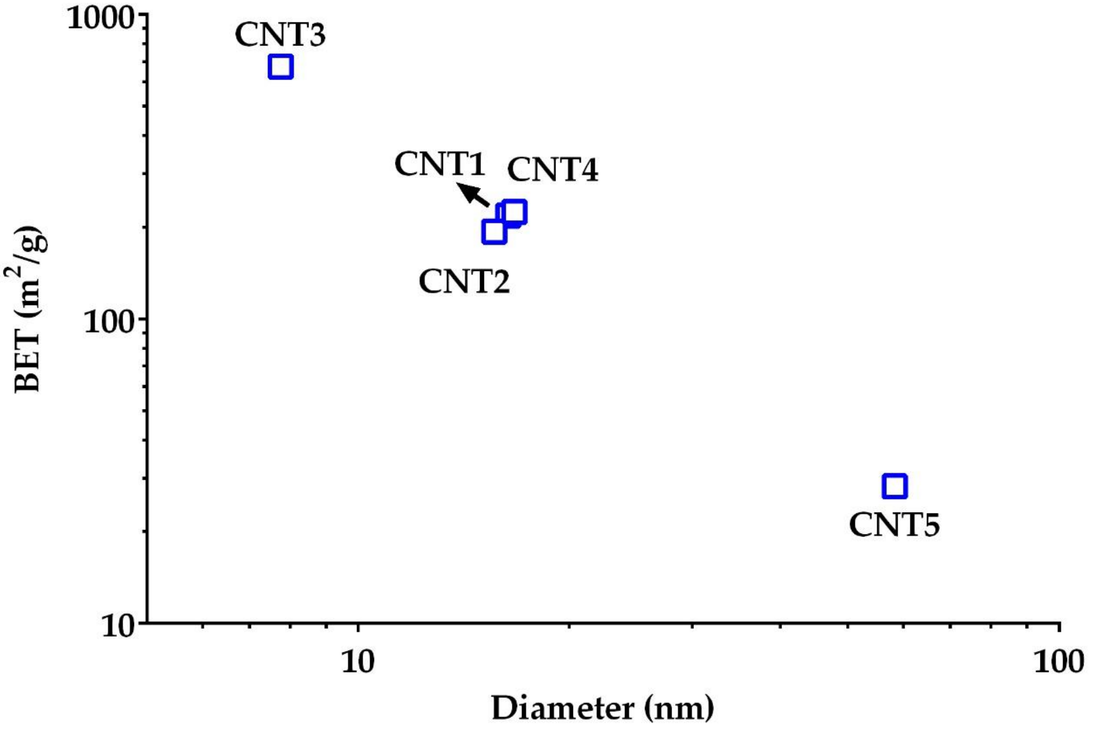

Figure S1. The correlation plot between the diameter of MWCNTs and BET. Note that both axes were expressed as log10 scale.

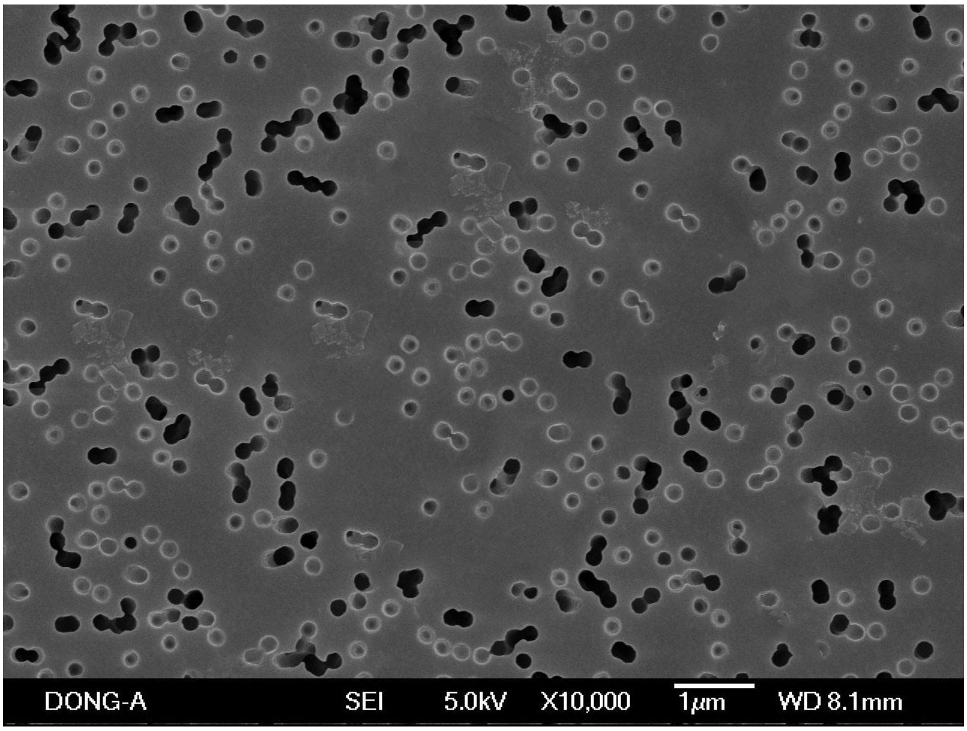

Figure S2. The representative SEM image of the undiluted MWCNT-free soluble fraction at 2.4 mg/mL. Note that the collected soluble fraction of MWCNTs showed the only pore of filter and salt crystals without any MWCNT fibers.

Table S1. The levels of selected elements (ppb) of soluble fractions of MWCNTs dispersed in ALF (pH 5.5).

Metals	Property	CNT1	CNT2	CNT3	CNT4	CNT5
Al	Other metals	12.9	11.6	112	9.49	7.14
As	Metalloids	0.1	0.1	0.1	0.1	0.1
B	Metalloids	0.105	0.099	0.082	0.082	0.087
Ba	Alkaline metals	0	0	0	0	0
Co	Transition metals	9.1	9.5	11.2	13.6	0
Cr	Transition metals	0.005	0.004	0.002	0.003	0.003
Cu	Transition metals	0.002	0.003	0.002	0.002	0.022
Fe	Transition metals	150.9	160.1	162	153.6	237
Ga	other metals	0	0.003	0	0	0
Li	Alkali metals	0.003	0.004	0	0.001	0.004
Mn	Transition metals	0	0	0	0	0
Mo	Transition metals	21.5	20.7	0.1	0.1	0.1
Ni	Transition metals	1.4	1.4	1.3	1.3	1.4
Rb	Alkali metals	0.021	0.037	0.033	0.037	0.034
Sb	Metalloids	0.009	0	0	0	0.006
Se	Nonmetals	0.021	0.008	0	0.066	0.02
Sn	other metals	0.009	0	0.006	0.003	0.007
Sr	Alkaline metals	0.011	0.013	0.011	0.013	0.012
Ti	Transition metals	0.001	0.001	0.001	0.001	0.001
W	Transition metals	0.014	0.003	0.008	0.008	0.004
Zn	Transition metals	15.7	8.3	6.9	10.3	4
